# Improving Reading Skills Using a Computerized Phonological Training Program in Early Readers with Reading Difficulties

**DOI:** 10.3390/ijerph191811526

**Published:** 2022-09-13

**Authors:** Susanna Forné, Anna López-Sala, Roger Mateu-Estivill, Ana Adan, Xavier Caldú, Xavier Rifà-Ros, Josep M. Serra-Grabulosa

**Affiliations:** 1 Department of Psychiatry and Legal Medicine, Universitat Autònoma de Barcelona, 08193 Bellaterra, Spain; 2 Department of Neurology, Hospital Sant Joan de Déu, 08950 Esplugues de Llobregat, Spain; 3 Department of Clinical Psychology and Psychobiology, University of Barcelona, 08035 Barcelona, Spain; 4 Institute of Neurosciences, University of Barcelona, 08035 Barcelona, Spain; 5 Department of Cognition, Development and Educational Psychology, University of Barcelona, 08035 Barcelona, Spain; 6 Cognition and Brain Plasticity Unit, Bellvitge Biomedical Research Institute, 08908 L’Hospitalet de Llobregat, Spain

**Keywords:** reading difficulties, remediation, dyslexia, computerized intervention, phonological training, early readers

## Abstract

In the last years, there has been a big effort to identify risk factors for reading difficulties and to develop new methodologies to help struggling readers. It has been shown that early intervention is more successful than late intervention, and that intensive training programs can benefit children with reading difficulties. The aim of our study is to investigate the effectiveness of an intensive computerized phonological training program designed to improve reading performance in a sample of children with reading difficulties at the early stages of their reading learning process. Thirty-two children with reading difficulties were randomly assigned to one of the two intervention groups: RDIR (children with reading difficulties following a computerized intensive remediation strategy) (*n* = 20) (7.01 ± 0.69 years), focused on training phonemic awareness, decoding and reading fluency through the computational training; and RDOR (children with reading difficulties following an ordinary remediation strategy) (*n* = 12) (6.92 ± 0.82 years), which consisted of a reinforcement of reading with a traditional training approach at school. Normal readers (NR) were assigned to the control group (*n* = 24) (7.32 ± 0.66 years). Our results indicate that both the RDIR and RDOR groups showed an increased reading performance after the intervention. However, children in the RDIR group showed a stronger benefit than the children in the RDOR group, whose improvement was weaker. The control group did not show significant changes in reading performance during the same period. In conclusion, results suggest that intensive early intervention based on phonics training is an effective strategy to remediate reading difficulties, and that it can be used at school as the first approach to tackle such difficulties.

## 1. Introduction

Learning to read is a complex process. It implies the interaction of at least visual, verbal, and attention systems. In addition to the mechanical aspects of reading, comprehension depends on at least 6 components: morphological awareness, language comprehension, fluency, oral vocabulary knowledge, real word decoding, and working memory [1].

Over the past two decades, an effort has been made to understand the neurobiology of reading, from birth to adulthood [2]. The National Reading Panel [3] meta-analysis results stress the importance of being able to adequately decode words during the initial phases of reading to become a good reader. More specifically, in second grade, at approximately age 7, children usually transit from ‘learning to read’ to ‘reading to learn’ [4].

Multiple studies have proven that primary school children with reading difficulties are prone to continue struggling with reading throughout subsequent years [5]. Therefore, in recent years, there have been significant efforts to identify the risk factors behind reading difficulties [6] and to develop new methodologies and intervention programs to help struggling readers [7,8,9]. In addition to explicit phonological processing skills, including phonological awareness, reading speed and accuracy, and their predictive role in reading performance, phonological skills of an implicit nature, such as long and short-term memory, working memory, or quick access to stored representations are other risk factors that work as a predictive value in the acquisition of reading skills [10]. Other skills, such as rapid automatized naming (RAN), have been found to predict reading speed and fluency [11]. Moreover, reduced verbal fluency skills in early childhood could also be predictive of dyslexia [12].

Regarding the remediation of reading difficulties, several studies have shown that early intervention is more successful than late intervention [13]. In this sense, phonics, including interventions that systematically teach letter-sound correspondences and decoding strategies, is the most researched treatment; it has proven to promote significant reading and spelling improvements in early reading stages [14]. In children and adolescents, phonics is the most effective approach to reading difficulties (see Galuschka and col. for a review) [15]. The importance of phonological processes-training during childhood has also been seen in languages with transparent spelling, as in the case of Spanish [9] or Finnish [16].

One approach to early detection and remedial reading is the Response to Intervention (RTI) approach [17]. Response to Intervention is a multi-tier approach for early identification and support of students with learning and behavior needs. In schools, when implementing RTI models, the most targeted academic area begins with reading. With the RTI approach, students are provided with evidence-based classroom reading instruction and supplemental intervention when needed; intervention decisions are based on student assessment data [18]. Even though there is major support for the RTI approach [7,18], it continues to be poorly implemented in Spain [19,20]. Recently, a study performed in the Canary Islands has shown the effectiveness of RTI in early identification and remediation of reading and math difficulties [21].

On the other hand, in terms of remedial interventions, it is interesting to highlight that, in recent years, computer-assisted training programs have been developed for reading difficulties. One such program is GraphoGame [22]. Initially, it was developed as a technology-based intervention method to aid children with reading difficulties. Today, the game is available to all Finnish school children as literacy support, and it has been implemented in over 20 countries as a reading instruction method [23]. Other studies have shown similar results after applying computerized training programs [24,25,26]. Additionally, several studies have also concluded that intensive remediation strategies based on action video games improve reading abilities and attention [27,28,29].

In Spain, a country where several languages are spoken in various regions in addition to Spanish, there are two studies showing the effectiveness of computer-assisted training programs. One of them was performed by Jiménez and col. [9] using a sample of 83 dyslexic Spanish children between 7.1 and 10.6 years of age. The authors of this study showed that phonological training using a speech-based computer-remediation program improved word decoding in dyslexic children. Recently, another study was carried out in Catalonia, a region in northeastern Spain, where Catalan and Spanish are both official languages [30]. This study used the “Binding Method” software, a method that was developed at the University of Barcelona to help children with reading difficulties so that they acquire and improve their reading skills, as it is described elsewhere [30]. In this study, the authors selected a sample of 347 children from 33 schools where Catalan was used as the principal language; they observed that all primary school children in 1st grade benefited from the follow-up of intensive phonological training using the Binding Method as an educational method to stimulate reading throughout the academic year. In this study, group and individual sessions were combined, and the results showed that the experimental group (Binding group) obtained better results in reading fluency (speed and accuracy) in all tests administered when compared to the control group at the end of the course. However, a comparative analysis was not carried out between children with reading difficulties and the rest of the children, nor was the efficiency of the Binding Method compared to an ordinary remedial program at the participating schools.

Thus, and to our knowledge, there are no studies assessing the effects on reading and writing of an intensive computer-training program in a Catalan sample of early readers with reading difficulties. Catalan, as is the case of Spanish, French, Italian, and Portuguese, is a romance language; it is spoken in Catalonia, with approximately 7.5 million inhabitants, and has a moderately transparent spelling [31].

The aims of our study were: (1) first, to analyze changes in reading fluency and accuracy, comprehension and spelling, in a group of early readers (6–7-year-old) with reading difficulties after following an intensive phonological-based computerized intervention program (the Binding method) which has been successfully applied to increase the reading performance in early readers [30]; (2) secondly, compare the benefits of this intensive program to the ordinary remedial program existing in the public schools of Catalonia. According to this recent study and other previous studies using computerized tools to train children with reading difficulties [9,26,32,33,34], and based on the fact that early phonological intervention is the most effective approach [14], we hypothesize that children with reading difficulties at early stages of the reading process (6–7 years old) will improve their reading performance after an intensive computerized phonological training program, and that the intensive computerized training will be more effective than the ordinary remedial program existing in the public schools of Catalonia. 

## 2. Methods

### 2.1. Participants

All participants were native Catalan or Catalan-speaking children, from urban zones, from average socio-economic backgrounds, and who were attending different public schools. The sample included a group of normal readers (*n* = 24, NR group) (7.32 ± 0.66 years), a group with reading difficulties who followed a digital and intensive remediation program (*n* = 20, RDIR group) (7.01 + 0.69 years), and a third group with reading difficulties who received a standard intervention reading program (*n* = 12, RDOR group) (6.92 + 0.82 years). Participants were randomly assigned to the RDIR or RDOR group. The control group was matched for age and gender at the beginning of the study (NR group, mean age = 7.32 ± 0.66; RDIR group, mean age = 7.01 ± 0.69; RDOR group, mean age = 6.92 ± 0.82). Teachers performed the initial selection of children with reading difficulties. After that, the selected children were assessed following a standardized protocol which included an estimation of their intelligence quotient (IQ), reading and spelling skills, executive function, selective attention, rapid naming and switching, and working memory and behavior. Although initially the RDOR group was composed by 18 participants, six participants were excluded from the final analysis for different reasons: 2 of them moved to another school after 2 and 5 weeks of training, and 4 of them were sick for a long period.

Inclusion criteria for the RDIR and RDOR groups were established by having a score below 1.5 standard deviations in at least three reading subtests. Exclusion criteria included having an IQ below 85, history of chronic disorders or mental illness, not speaking or understanding Catalan, and having motor or sensory deficits that could affect their neuropsychological assessment.

After providing a complete description of the study to all participants, written and verbal informed consent was obtained from a parent and affirmed assent was obtained from the children. The research ethics committee Institutional Review Board (IRB00003099) of the University of Barcelona (Spain) approved the study.

### 2.2. Catalan Language

Catalan is a Romance language of the Western Romance group that has common features with other Ibero-Roman languages, such as morphology (especially nominal and verbal flexions), and to Galician-speaking languages (phonetics and, in part, lexicon), with much affinity to Spanish. Catalan is a language with a moderately transparent spelling. It is used to teach more than 1.5 million children. The Catalan vowel system has 5 letters that are represented by 8 sounds: /ə/, /a/, /e/, /ɛ/, /i/, /o/, /ɔ/, and /u/, and consonant phonemes, which, in some cases, do not have a direct correspondence with a grapheme. In fact, some sounds may be written in five different ways. For example, /b/- v or b (*veure* or *beure*), /p/- p or b (*pal* or *calb*), /g/- g or gu (*gol* or *guineu*), /t/- t or d (*pot* or *fred*), /k/- c, q or g (*casa*, *quilo* or *biòleg*), /z/- s o z (*rosa* or *zebra*), /ʃ/- x or ix (*feix* or *coixí*), /s/- s, ss, ç, c, sc (*sopa, cassola, peça, cirera, piscina*), / tʃ͡t/- ig or –g (*faig* or *mig*), / dʒ͡/- tj, tg, dj (*platja, fetge, adjectiu*), among other sounds.

### 2.3. Neuropsychological Assessment

All participants in the study were assessed individually, both before and after the training program. A trained neuropsychologist (S.F.) conducted the assessment. After the initial evaluation, the three groups started the ‘intervention’ period which lasted 16 weeks: the RDIR group began the phonological training. The RDOR group followed a standard remedial reading support program at school while the normal readers received no intervention of any type. After the training period, children were re-assessed using the same evaluation protocol, except for the IQ estimation.

### 2.4. Measures 

*IQ estimation.* The WISC-IV Vocabulary subtest [35] was used to obtain an estimation of verbal IQ (VIQ), and the Block design subtest was used to obtain an estimation of the performance IQ (PIQ).

*Attention/verbal short-term memory.* This measure was assessed by Digit span (WISC-IV) [35]. The task was to repeat sequences of digits (spanning from two to eight digits) in the correct order. Each correctly repeated span was scored.

*Working memory.* This measure was also assessed using Digit span (WISC-IV) [35]. The task was to repeat digits (spanning from two to eight digits) backwards in the correct order. Each correctly repeated span was scored.

*Phonetic and semantic fluencies.* These measures were used to assess executive function and verbal fluency. First, children were asked to generate words that began with letters FAS in a 60-second interval per letter (the total number of words was used as a measure for verbal phonetic fluency). Afterwards, the children were asked to generate as many names of animals as possible within a one-minute interval (total number of names was used as a measure for semantic verbal fluency) [36].

*Naming speed task.* Rapid automatized naming of letters and colors was used as a measure for naming speed [37]. The total time in seconds for naming letters and colors was registered for each child. 

*Reading.* This measure was assessed by the standardized Catalan reading skills tests TALE-C [38] and PROLEC-R [39]. Measures for reading speed and accuracy were obtained using the TALE-C letters, syllables, words and text subtests, and the pseudowords measures by the PROLEC-R. Text comprehension was assessed using the TALE-C text comprehension test. 

*Spelling.* Natural and arbitrary orthography were measured using the TALE-C writing subtest [38].

### 2.5. Intervention

#### 2.5.1. Digital and Intensive Remediation Program 

The digital and intensive remedial reading program was based on the application of the “Binding Method” software (University of Barcelona, Barcelona, Spain, 2014). This method was developed to help children with reading difficulties acquire and improve their reading skills (University of Barcelona Department of Basic Psychology, in collaboration with the Josep Finestres Foundation, at https://www.ubinding.cat (accessed on 17 August 2022)). The Binding Method was developed following the Response to Intervention Model, which is intensive and based on the progress of each child [40]. It provided training in a variety of tasks. These included: (1) Reading speed, with exercises in which children are asked to read syllable lists, invented or real words as quickly as possible, while measuring the effectiveness and the time it takes to do that task; (2) Phonological awareness, with tasks in which: (a) children had to indicate images on a board from among those that begin with a specific letter; (b) look for words from a given sound-set; (c) suppress sounds; or (d) invert syllables; (3) Short-term verbal memory, with the presentation of several syllables or words that should be read aloud and once these disappeared from the screen, repeating them in sequential order; (4) Working memory, with activities in which there were also several syllables and words to be read and memorized, but only the one that appeared in a specifically requested position or with sequences of syllables where the child had to decide whether or not the new word appearing on the screen was equal to the previous one (1-back task); (5) Vocabulary, with tasks that should be noted from among a large group of both real and invented words, but they only chose real words. A remediation program consisted of a 16-week intervention, with four sessions per week, and 15 minutes per session. A special education teacher, previously instructed on how to use the training program, supervised the training sessions. The children’s answers were digitally registered as “correct” or “incorrect” by the computer according to the teacher’s assessment. Two psychologists (JMSG and JMSS) selected the daily training tasks based on each child’s reading level (accuracy and speed-reading scores from previous sessions) and knowing that the program has all the exercises labeled according to the complexity of the task. Accuracy was calculated from the total number of hits for each task (i.e., good reading of the word/pseudoword). Speed reading was also calculated as the amount of time between the presentation of a word/pseudoword and its reading. Performance was categorized as optimal if the hits were above 75%. The two psychologists selected the training task based on the performance of each subject. Thus, only the level of difficulty was increased from a yield of 75% or higher in the previous level. Tasks were classified according to their complexity: monosyllables, bisyllables, trisyllables, and the combinations of vowels and consonants, depending on the probability of finding those combinations in the Catalan language.

The intervention used in the RDIR group had not previously been used in any of the selected schools. Each session began with the children performing a speech rate activity, training pairs of words for 2 minutes. A 15-minute phonological training session started with a combination of phonological awareness, phoneme decoding, and reading fluency activities (more than 50% of the activities), together with verbal working memory tasks. These tasks adapted the level of difficulty to each child. 

Phonological awareness activities consisted of letters and syllables identification (both words and pseudowords), quick letter naming, rhymes identification, and segmentation of words into syllables. Pictures accompanied certain activities, in which the child had to decide those words with the same initial phoneme as the picture.

Decoding and reading fluency activities used a bottom-up strategy where each child read groups of words and pseudowords with different structures: consonant-vowel (CV), vowel-consonant (VC), consonant-vowel-consonant (CVC), consonant-consonant-vowel (CCV), consonant-consonant-vowel-consonant (CCVC), and the combination of these structures in bisyllabic and trisyllabic words.

Words and pseudowords were displayed on the screen using a variety of formats: one-by-one in the center, as a dynamic list where a white marker signaled the word to be read, or as a game where the child played driving a racecar or riding a horse. In this case, the second player was controlled by the program, which decided the speed of the figure (car or horse) depending on the level of each child in the previous race. At the end of each session, each child was asked to score the difficulty and the extent to which they enjoyed the session.

#### 2.5.2. Ordinary Remediation Program

This intervention consisted of reinforcing reading (speed, precision, and comprehension) at school. The characteristics of the RDOR group were determined by what is the standard practice at public schools in Catalonia. The work was undertaken in small groups (four children), with a frequency of one day per week during 16 weeks, 60 minutes per session, with a variety of activities related to reading aloud or silently, writing, phonological awareness and reading comprehension. Training sessions were less systematic than intensive intervention sessions, carried out by a special education teacher who decided which activities were to be included in each session.

### 2.6. Statistical Methods

Statistical analyses were carried out in the SPSS software, version 26 (IBM Corp, New York, NY, USA, 2019). For the comparison of demographic characteristics of the samples, independent samples t-tests and chi-square tests were used.

A mixed design ANOVA with 3 groups (group: two kinds of intervention and one control condition) and 2 occasions (time: pre-test vs. post-test) was used to test for differences in the effectiveness of the treatment. After that, the comparison of the distribution for quantitative-type variables was carried out using the ANOVA test for independent samples (to compare the three study groups) since the normality and equality of variances criteria were met. In each group, a repeated-measures analysis of variance was performed to study the effects of the intervention. Cohen’s d effect sizes were reported. In case of violations of sphericity, Greenhouse–Geisser corrected F were reported. Partial η2 values and Cohen’s d effect sizes were reported. Effect sizes for Cohen’s d are interpreted as large at a 0.99 cutoff, medium at 0.57, and small at 0.25. Partial eta square (η2) effect size are interpreted as small effects from η2  =  0.01 to η2  ≤  0.06, as medium effects from η2  >  0.06 to η2  <  0.14, and as large effects from η2  ≥  0.14 [41].

## 3. Results

All participants had an estimated IQ in the normal range. Regarding the literacy variables, *group x time* interaction was significant for all variables assessed in speed reading, reading accuracy, and spelling categories.

Post-hoc tests showed an improvement in both RDIR and RDOR groups for reading and spelling at 16 weeks after starting the training. This improvement was higher in the RDIR than the RDOR group. Results of the training effect analyses are shown in Table 1. While children in the computerized and intensive remediation program increased their performance in all of the reading and spelling variables (except for arbitrary orthography), the ordinary intervention group showed a slight performance increase after intervention only for speed of word and text reading, and in the accuracy of syllable, word, and text reading. However, in the NR group, the results showed no differences in performance between the first and second assessment.

In the case of the multiple comparisons, Table 2 shows the performance gain for each group when comparing the results for the reading and spelling tests at pre- (T1) and post-intervention (T2). These results showed a higher effectivity of the RDIR group than the RDOR group. In this sense, comparison between RDIR and NR groups showed that RDIR had a significantly higher gain than NR group in all speed and accuracy reading measures (except for letter reading accuracy, text comprehension, and arbitrary orthography). On the other hand, comparison between RDOR and NR groups showed that the RDOR group only had a significantly higher gain than the NR group in text speed reading, text comprehension, and natural orthography. Finally, the comparison between RDIR and RDOR groups showed that the RDIR group had a significantly higher gain than the RDOR group in speed reading of words and pseudowords, and in letter, word, pseudoword, and text measures of reading accuracy.

Moreover, post-intervention comparisons showed that although there was a significant improvement, RDIR and RDOR continued to perform below the NR group (Table 3). 

The comparison between the RDIR and RDOR groups at the post-intervention period indicates that the first group outperformed the RDOR group in word reading accuracy, and in five measures for reading speed: letters, syllables, words, pseudowords and text, and in arbitrary spelling.

In terms of the implicit factors that contribute to reading, performance was assessed in short-term memory measures, working memory, verbal fluency, and rapid naming, all considered phonological abilities of an implicit nature that also influence the acquisition of reading skills [10] (Defior, 2011). In this sense, we observed that the NR group obtained a performance in the normal range in T1 and T2 assessments. The RDIR group obtained a significant increasing of performance in four of the six variables assessed: RAN objects (T1: 40.90 ± 7.03; T2: 43.70 ± 8.97; *p* = 0.036; d = 0.36), RAN colors (T1: 33.80 ± 7.08; T2: 38.20 ± 8.02; *p* < 0.001; d = 0.60), semantic verbal fluency (T1: 42.80 ± 8.37; T2: 58.60 ± 12.85; *p* < 0.001; d = 1.49), and direct digits (T1: 43.75 ± 5.02; T2: 48.55 ± 5.20; *p* = 0.001; *p* = 0.96), while the RDOR group failed to obtain significant differences in any of the values measured.

## 4. Discussion

The aim of our study was to analyze changes in reading fluency and accuracy in a group of early readers with reading difficulties after following an intensive computerized remediation program, and to compare these changes with those obtained by an ordinary remediation program, which is the standard program at public schools in Catalonia. Results showed that the children in the intensive remediation program had a bigger improvement in all variables for reading accuracy, speed and natural spelling when compared with the children who followed an ordinary remediation program, whose improvement was weaker.

The Importance of preventing and detecting reading difficulties in children fosters the interest of the scientific community to know the best moment for intervention, and how this should be undertaken. Our results are congruent with previous studies indicating that early remediation (6–8 years old) [24,42] and phonics instruction [3,30,43] yield a higher benefit for reading skills than later remediation, and the combination of both is the most effective approach for children with learning disabilities when learning to read and spell [15]. Our results also coincide with previous studies, indicating that remediation intervention should include bottom-up training at the early stages of the reading learning process [44]. Likewise, our results positively support the work performed within the framework of RTI methodology. In this sense, this work complements a recent study that has been found to significantly improve reading and learning among children who followed a RTI program that used the Binding Method [30]. Projects like this could be the basis for contributing to the implementation of RTI in Spain, which is a country where it still has limited presence [20,21]. It is now known that, in addition to facilitating reading in primary school children, the Binding Method significantly improves reading mechanics in children with reading difficulties. 

In our case, the benefit of intervening in the RDIR group had an effect size mean value of 1.05 for speed reading/reading fluency, 1.13 for reading accuracy, and 0.76 for natural spelling. These results are in keeping with the meta-analysis by Ehri and col. [43], who proposed an overall statistically significant positive effect size for phonics instruction of reading. The results obtained in our study are in line with studies carried out with more transparent spellings, which, apart from working on phonological skills, also affect the fluency of reading. This latter aspect is one of the variables that is a major obstacle in surface languages [45]. However, it is essential to consider that spelling in Catalan is not as transparent as Finnish or Spanish [31]. In this sense, almost 100% of all Finnish letters have only one reading and nearly 90% only one spelling; in the case of Spanish, 96% of the letters have only one reading and 90% only one spelling; in Catalan, 76% of the letters have only one reading and 70% only one spelling.

When it comes to implicit phonological skills, the RDIR group also showed greater improvement after intervention; this group obtained significant and greater changes in comparison with the rest for RAN measurements, verbal fluency, and attention/working memory. The execution of RAN tasks is highly predictive of success in learning to read and write transparent spellings [46]. Moreover, early levels of naming speed have been related to predicting future reading skills [47].

In our study, the RDIR group achieved mean scores in all reading speed values, although these were still lower than those of the NR group, thus indicating the need for more continuity in the intervention.

As mentioned previously, early identification and preventive intervention is essential to reduce reading difficulties seen in many elementary students and to ensure that they receive effective remediation [24]. As C.A. Denton [18] (2012) indicated, there has been an increase in educational initiatives aimed at preventing reading difficulties before age 8. It is imperative that early detection be carried out in schools and that the reading support programs implemented be effective in helping these children. The results obtained in our study coincide along these same lines. Upon analyzing how the intervention type received by children with reading difficulties influenced their performance for speed, accuracy, reading comprehension and spelling tasks, we found that those who followed the computer training program (RDIR) obtained better results than those who received ordinary school support (RDOR). In Catalonia, public schools offer specific small-group support to those students who need to reinforce their reading acquisition skills in a one week session. Thus, the results obtained in our study endorse and support the need to rethink what type of intervention schools offer, and also to work within a model of good practice when teaching to read so that this focuses on the application of the more effective support programs; if not for all, at least for the majority of children with difficulties in this area [48].

Moreover, several studies have shown that computer-aided learning is an attractive and effective method for improving learning in children with reading problems. The results of a meta-analysis [49] suggest that digital programs are more effective than conventional instruction methods in terms of learning. It is interesting to highlight that in recent years, there has been an increase of studies using computer-assisted training programs to remediate reading disabilities which have been shown to be beneficial for early readers with difficulties [9,16,22,26,33,34]. One example of this is the GraphoGame computer game, developed as a way to train letter-sound connections and reading skills; the game has shown to have a positive effect on children with reading difficulties [50]. As indicated in some of the aforementioned studies, for an intervention to be effective, it must offer phonological training, whether individually or in small groups, while at the same time being repetitive, intensive (between 4 and 5 sessions per week), and motivating [51]. The computer training applied in our study is characterized by combining all of these variables.

Finally, it is important to highlight certain limitations of our study. First of all, the sample size of the RDOR group (*n* = 12) was smaller than the sample of the RDIR (*n* = 20) and the NR (*n* = 24) groups. It cannot be ruled out that the differences observed between treatments could be due, in part, to these differences in group sizes. On the other hand, the fact that during the pre-treatment there were differences in the explicit and implicit phonological processing skills, with slight differences between the RDIR and RDOR groups, may have influenced the results of the pre- and post-treatment comparisons between groups. Thus, future studies need to take these limitations into account by using more homogeneous samples. Moreover, future studies should follow the children who receive intensive interventions further in time to see if there are some children who plateau in their improvement or if some are able to catch up with their peers.

In conclusion, our results indicate that children with reading difficulties who receive an early computerized and intensive training program, based on phonemic awareness, decoding, and reading fluency exercises, significantly improve their performance in precision, fluency, reading comprehension, and spelling.

## Figures and Tables

**Table 1:** Performance on the reading test for each intervention group before and after intervention.

	NR (*n* = 24)		RDIR (*n* = 20)		RDOR (*n* = 12)			Interaction Group x Time	
	Mean (s.d.) Time 1 Time 2	Effect Size	Mean (s.d.) Time 1 Time 2	Effect Size	Mean (s.d.) Time 1 Time 2	Effect Size	F	*p*	*η_p_* ^2^
SPEED READING									
Letters	61.79 (2.67) 62.54 (4.23)	0.22	51.35 (9.35) *** 59.45 (6.22)	1.05	46.92 (9.98) 49.75 (10.38)	0.29	5.285	0.008	0.166
Syllables	60.63 (2.22) 61.29 (2.65)	0.28	54.50 (5.23) *** 59.85 (3.88)	1.19	50.50 (6.24) 52.33 (8.46)	0.26	4.863	0.012	0.155
Words	59.96 (3.42) 60.67 (3.46)	0.21	39.65 (6.24) *** 55.30 (4.69)	1.05	38.50 (11.81) 43.17 (9.88) **	0.45	14.04	0.001	0.346
Pseudowords	51.13 (7.83) 53.04 (8.82)	0.23	31.10 (9.07) *** 45.40 (11.94)	1.38	27.83 (7.78) 30.33 (10.01)	0.29	14.50	0.001	0.354
Text	59.13 (3.89) 59.71 (3.8)	0.15	46.80 (11.54) *** 54.05 (6.26)	0.80	42.83 (8.57) 45.33 (7.04)	0.33	6.02	0.004	0.185
Text Comprehension	59.33 (4.39) 58.79 (2.21)	0.16	47.65 (8.41) *** 53.80 (6.60)	0.83	47.17 (4.11) * 50.25 (4.83)	0.72	10.11	0.001	0.276
READING ACCURACY									
Letters	58.83 (3.75) 59.33 (3.61)	0.14	44.00 (9.06) ** 49.45 (6.24)	0.72	47.67 (7.19) 44.00 (8.78)	0.48	5.14	0.007	0.170
Syllables	63.25 (3.99) 64.83 (2.22)	0.5	49.80 (8.79) *** 56.00 (7.07)	0.80	53.08 (7.98) * 57.33 (8.73)	0.53	3.66	0.032	0.121
Words	62.00 (3.01) 62.63 (3.27)	0.2	38.25 (6.20) *** 48.95 (7.45)	1.60	39.33 (8.64) * 42.58 (5.11)	0.48	18.70	0.001	0.414
Pseudowords	52 (7.44) 53.67 (6.58)	0.24	22.55 (4.59) *** 33.55 (13.02)	1.16	30.08 (8.67) 32.25 (10.45)	0.24	5.65	0.006	0.176
Text	59.88 (1.92) 60.21 (2.26)	0.16	34.65 (11.14) *** 53.20 (9.54)	1.84	36.67 (10.29) *** 46.75 (8.98)	1.09	23.83	0.001	0.473
Text comprehension (hits)	53.38 (10.41) 56.25 (10.55)	0.57	41.50 (10.21) ** 47.40 (7.51)	0.68	47.08 (9.18) 43.08 (7.55)	0.5	6.18	0.004	0.189
SPELLING									
Arbitrary Orthography	59.29 (8.13) 61.50 (7.47)	0.29	54.60 (7.37) 53.65 (7.31)	0.13	43.92 (10.94) 44.42 (12.04)	0.05	1.36	0.266	0.049
Natural Orthography	63.67 (5.10) 62.13 (4.04)	0.34	39.15 (11.69) *** 47.85 (11.91)	0.76	39.17 (6.09) 42.17 (7.02)	0.48	7.74	0.007	0.226

*Note.* NR: control group; RDIR: group with reading difficulties who followed a digital and intensive remediation program; RDOR: group with reading difficulties who received a standard reading intervention program. * *p*-value ≤ 0.05; ** *p*-value ≤ 0.005; *** *p*-value ≤ 0.001.

**Table 2:** Comparison of performance gain after the training period.

				NR–RDIR		NR–RDOR		RDIR–RDOR	
	NR	RDIR	RDOR	t–Student	*p*	t–Student	*p*	t–Student	*p*
SPEED READING									
Letters	0.75 ± 4.29	8.10 ± 10.58	2.83 ± 6.58	−2.91	0.008	−1.14	0.261	1.548	0.132
Syllables	0.667 ± 1.46	5.35 ± 5.38	1.83 ± 8.26	−3.77	0.001	−0.48	0.637	1.462	0.254
Words	0.70 ± 1.23	15.65 ± 6.34	4.66 ± 8.18	−4.43	<0.001	−2.26	0.044	3.994	<0.001
Pseudowords	1.91 ± 5.33	14.30 ± 11.84	2.08 ± 4.75	−4.32	<0.001	−0.09	0.928	3.39	0.002
Text	0.58 ± 1.79	7.25 ± 8.78	2.50 ± 7.64	−3.33	0.003	−0.85	0.409	1.551	0.131
Text Comprehension	−0.54 ± 3.68	6.15 ± 6.32	3.08 ± 4.44	−4.17	<0.001	−2.60	0.014	1.47	0.152
READING ACCURACY									
Letters	0.50 ± 3.20	5.45 ± 10.71	3.66 ± 8.48	−1.99	0.059	−1.643	0.125	2.51	0.018
Syllables	1.58 ± 3.54	6.20 ± 7.01	4.25 ± 6.60	−2.67	0.013	−1.30	0.212	0.77	0.443
Words	0.62 ± 1.86	10.70 ± 6.06	3.25 ± 8.75	−7.15	<0.001	−1.02	0.325	2.84	0.008
Pseudowords	1.66 ± 5.78	11.00 ± 14.89	2.16 ± 2.94	−2.64	0.014	−0.34	0.733	2.56	0.018
Text	0.33 ± 2.69	18.55 ± 12.15	10.08 ± 9.88	−6.56	<0.001	−3.35	0.006	2.04	0.050
Text comprehension (hits)	2.87 ± 8.68	5.90 ± 9.34	4.00 ± 7.08	−0.01	0.99	−1.610	0.184	3.38	0.002
SPELLING									
Arbitrary Orthography	2.20 ± 6.28	−0.95 ± 8.75	−1.75 ± 9.20	1.39	0.172	1.52	0.138	0.24	0.808
Natural Orthography	−1.54 ± 4.49	8.70 ± 13.06	3.00 ± 4.41	−3.34	0.003	−2.87	0.007	1.78	0.086

*Note.* NR: control group; RDIR: group with reading difficulties who followed a digital and intensive remediation program; RDOR: group with reading difficulties who received a standard reading intervention program.

**Table 3:** Comparison of performance at post-intervention.

				NR–RDIR		NR–RDOR		RDIR–RDOR	
	NR	RDIR	RDOR	t–Student	*p*	t–Student	*p*	t–Student	*p*
SPEED READING									
Letters	62.54 ± 4.23	59.45 ± 6.22	49.75 ± 10.38	2.11	0.04	4.10	0.001	3.16	0.004
Syllables	61.29 ± 2.65	59.85 ± 3.88	52.33 ± 8.46	1.82	0.076	5.44	<0.001	2.78	0.015
Words	60.67 ± 3.46	55.30 ± 4.69	43.17 ± 9.88	4.11	<0.001	5.71	<0.001	4.80	<0.001
Pseudowords	53.04 ± 8.82	45.40 ± 11.94	30.33 ± 10.01	7.70	<0.001	6.48	<0.001	3.47	0.002
Text	59.71 ± 3.80	54.05 ± 6.26	45.33 ± 7.04	3.43	0.001	6.61	<0.001	3.92	<0.001
Text Comprehension	58.79 ± 2.21	53.80 ± 6.60	50.25 ± 4.83	5.66	<0.001	5.83	<0.001	1.67	0.105
READING ACCURACY									
Letters	59.33 ± 3.61	49.45 ± 6.24	44.00 ± 8.78	5.94	<0.001	7.46	<0.001	2.07	0.47
Syllables	64.83 ± 2.22	56.00 ± 7.07	57.33 ± 8.73	6.21	<0.001	2.92	0.013	−0.53	0.596
Words	62.63 ± 3.27	48.95 ± 7.45	42.58 ± 5.11	7.72	<0.001	14.32	<0.001	2.43	0.021
Pseudowords	53.67 ± 6.58	33.55 ± 13.02	32.25 ± 10.45	5.93	<0.001	7.66	<0.001	0.42	0.673
Text	60.21 ± 2.26	53.20 ± 9.54	46.75 ± 8.98	2.93	0.008	5.11	<0.001	1.97	0.058
Text comprehension (hits)	56.25 ± 10.55	47.40 ± 7.51	43.08 ± 7.55	3.03	0.004	3.84	0.001	1.55	0.130
SPELLING									
Arbitrary Orthography	61.50 ± 7.47	53.65 ± 7.31	44.42 ± 12.04	3.32	0.002	5.25	<0.001	2.72	0.011
Natural Orthography	62.13 ± 4.04	47.85 ± 11.91	42.17 ± 7.402	4.79	<0.001	10.87	<0.001	1.70	0.100

*Note.* NR: control group; RDIR: group with reading difficulties who followed a digital and intensive remediation program; RDOR: group with reading difficulties who received a standard reading intervention program.

## Data Availability

The data from this study are available from the authors upon request.
